# Exploring the impact of online education on student engagement in higher education in post-COVID-19: what students want to get?

**DOI:** 10.3389/fpsyg.2025.1574886

**Published:** 2025-07-09

**Authors:** Ziqi Deng, Zhi Yang

**Affiliations:** ^1^School of Continuing Education, Nanjing University of Science and Technology, Nanjing, Jiangsu, China; ^2^Griffith Business School, Griffith University, Southport, QLD, Australia

**Keywords:** student engagement, online education, higher education students, post-COVID-19, teaching and learning

## Abstract

The sudden outbreak of COVID-19 has led to an unprecedented impact on traditional higher education classrooms. To ensure that students can continue receiving quality education, online learning has become a mainstream mode of instruction. Therefore, increasing student engagement become a key priority for teachers in online teaching contexts. Few reviews examine student engagement in education in the post-COVID-19 era. To address this gap, the present study aims to explore the key factors that influence student engagement in classroom settings in this context. We identified 30 papers related to our research focus from 498 articles retrieved from the Web of Science and Scopus databases, following the 2020 PRISMA framework. After reviewing related studies, this study examined the characteristics of student engagement about cognitive, affective, and behavioral dimensions. We also analyzed the impact of online education on student engagement. Our findings suggest that emotional, cognitive, and behavioral engagement are interconnected and influence one another. In addition, teachers’ support for students’ cognitive and emotional needs plays a vital role in fostering their behavioral engagement. This article can help educators better understand the definition of engagement and the factors that influence student engagement in the classroom.

## 1 Introduction

With the outbreak of COVID-19, face-to-face teaching stopped in many universities. Online education has become the first choice for higher education teachers (Xinogalos, 2022). Several studies have focused on the development of online education during the COVID-19 period (Chen et al., 2021; Limniou et al., 2022; Lu et al., 2022). However, many teachers and students have continued to face challenges under the current situation (Walker and Koralesky, 2021). One of the challenges in online learning was that students may lack intrinsic motivation, which can increase the risk of dropout (Northey et al., 2015).

Many researchers have highlighted the importance of student engagement and explored its benefits and challenges (Jeong, 2023; Northey et al., 2018). The study by Chiu (2022) indicated that teachers’ instructional strategies can help shift students’ motivational orientation from extrinsic to intrinsic. To achieve this goal, school leaders may need to provide diverse online learning resources for students (Salta et al., 2022). Additionally, school leaders should also develop interesting learning materials to stimulate students engagement (Spitzer et al., 2021). Khlaif et al. (2021) noted that the quality of classroom content was a key factor influencing student engagement. Whereas, online learning may exacerbate digital inequality, which in turn negatively affects student engagement (Agung et al., 2020; Domina et al., 2021). Schools in some underdeveloped regions may lack essential online learning resources, which raises concerns about the equity and effectiveness of online education (Khlaif et al., 2021).

Since the COVID-19 pandemic, online teaching has evolved into a mainstream educational format (Palomino et al., 2023). During COVID-19, individuals, schools, and social institutions were affected in different ways (Ahshan, 2022). Similar examples abound in the literature (Cranfield et al., 2021). For instance, Stang-Rabrig et al. (2022) offered recommendations on the challenges and opportunities involved in reshaping online education during the COVID-19 pandemic. Since then, this article has sparked ongoing discussion and debate among researchers (Irena and Jolita, 2022).

With the development of online education, researchers have shown growing interest in student engagement. This study first provided a detailed interpretation of the definition of student engagement, and then investigated how online education affected it in the post-COVID-19 era. In this context, the following research questions were formulated:

- How is student engagement defined in the studies included in this review?
- What is the impact of online education on student engagement in the post-COVID-19?

By answering these two questions, we can better understand the advantages and challenges of online education and learning. Based on this understanding, researchers can better explore student engagement and develop targeted research questions. The teachers can better understand what students want and teach better in online lessons.

## 2 Methods

### 2.1 Search strategy

The process of article selection followed the Preferred Reporting of Items for Systematic Reviews and Meta-Analyses (PRISMA) Statement (Tricco et al., 2018). We searched Web of Science and Scopus on August 2nd, 2024 for peer-reviewed articles on online education and student engagement. We operationalized different permutations of each keyword based on previously validated searches. We drew on a series of reviews to identify keyword variants. The final keywords for the article were identified as student engagement (Northey et al., 2015), online education (Wang et al., 2021), higher education students (Vu et al., 2022), post-COVID-19 (Jeong, 2023), teaching and learning (Ahshan, 2022). For student engagement and Post-COVID-19, we also drew on the article of Addae (2023), which contained the standard expressions of student participation. For online education, we selected through multiple words comparisons from the articles of Vermeulen and Volman (2024) and Yuyun (2023) (Table 1).

**Table 1:** Terms used to search two databases related to research on student engagement.

Step	Terms	Results
Database: web of science		
1	TS = (“online education” OR “online learning” OR “virtual classroom” OR “distance learning” OR “remote learning” OR “m-learning” OR “mobile” OR “distance” OR “e-learning” OR “online and hybrid teaching and learning” OR “online distance learning” OR “distance education” OR “learning and teaching online”)	1,663,023
2	TS = (“student engagement” OR “learner engagement” OR “student involvement” OR “student participation” OR “interaction” OR “digital collaboration” OR “collaborative learning”)	308,243
3	TS = (“higher education students” OR “tertiary education students” OR “university students” OR “undergraduate students” OR “digital natives”)	100,234
4	TS = (“COVID-19” OR “coronavirus” OR “COVID19” OR “post-infectious” OR “post-recovery” OR “postviral” OR “Covid-19” OR “COVID-19 pandemic” OR “post-COVID- 19” OR “Post-Covid-19” OR “post-covid-19” OR “Post-COVID-19” OR “coronavirus disease pandemic”)	568,378
5	1 AND 2 AND 3 AND 4	269
Database: scopus		
1	TS = (“online education” OR “online learning” OR “virtual classroom” OR “distance learning” OR “remote learning” OR “m-learning” OR “mobile” OR “distance” OR “e-learning” OR “online and hybrid teaching and learning” OR “online distance learning” OR “distance education” OR “learning and teaching online”)	2,742,161
2	TS = (“student engagement” OR “learner engagement” OR “student involvement” OR “student participation” OR “interaction” OR “digital collaboration” OR “collaborative learning”)	5,214,743
3	TS = (“higher education students” OR “tertiary education students” OR “university students” OR “undergraduate students” OR “digital natives”)	147,466
4	TS = (“COVID-19” OR “coronavirus” OR “COVID19” OR “post-infectious” OR “post-recovery” OR “postviral” OR “Covid-19” OR “COVID-19 pandemic” OR “post-COVID- 19” OR “Post-Covid-19” OR “post-covid-19” OR “Post-COVID-19” OR “coronavirus disease pandemic”)	712,793
5	1 AND 2 AND 3 AND 4	591

We applied the fields title/abstract in the search. The full details are available in Appendix. Our initial search identified a total of 269 articles in Web of Science and 591 in Scopus, which were imported into Zotero reference management software. Of these 860 articles, 227 were identified as duplicates, leaving a total of 633 for screening and eligibility stages.

### 2.2 Inclusion and exclusion

**Figure 1:**
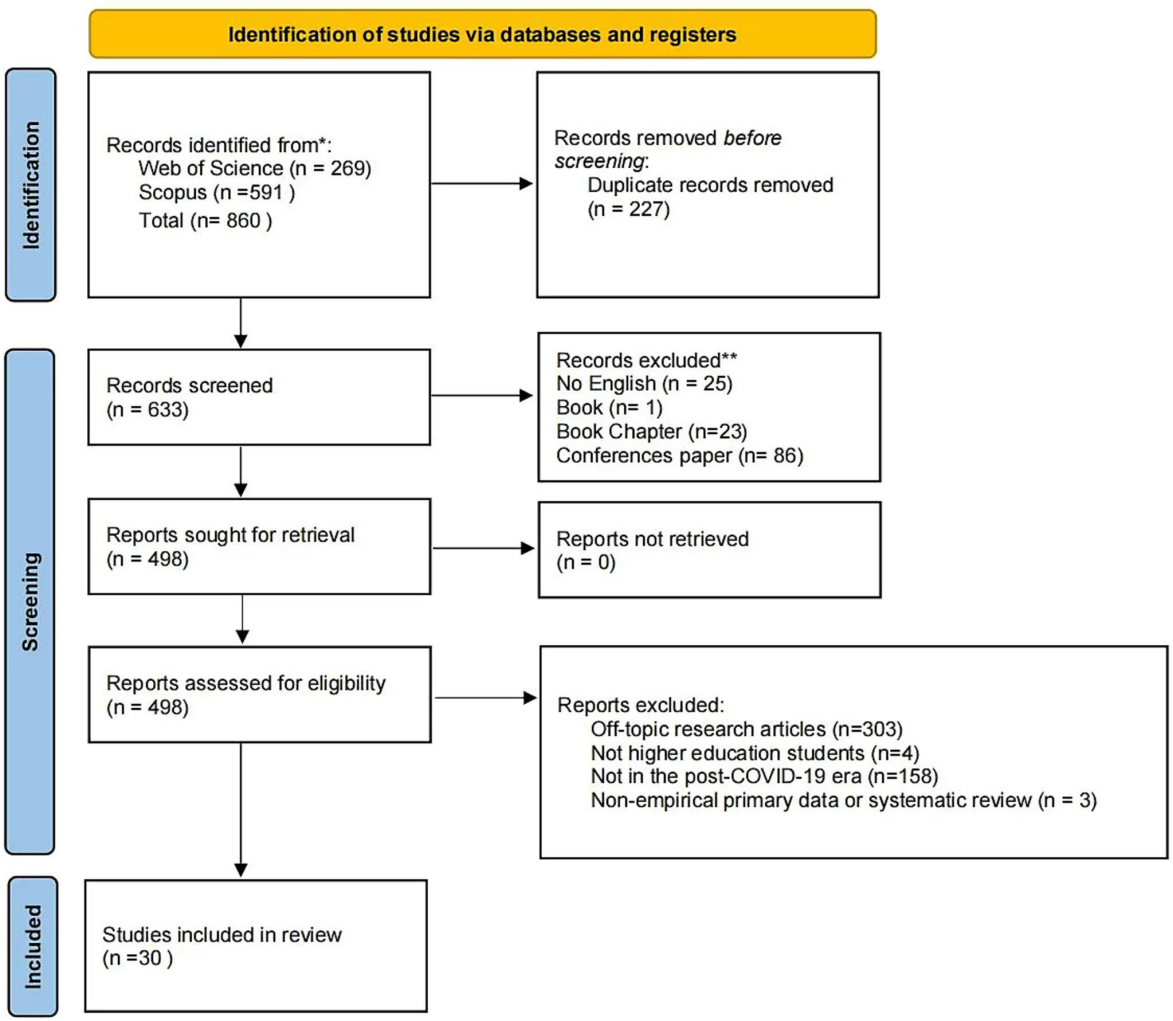
Process of inclusion/exclusion.

We applied a series of inclusion and exclusion criteria. Articles were included if they were: (i) written in English; (ii) published in peer-reviewed journal; (iii) empirical research article; (iv) research participation involved higher education students; (v) research about student engagement in online education; (vi) research period focused on post-COVID-19. They were excluded if the type of non-empirical primary data or dissertation, because this was not consistent with the purpose and significance of the research. Of the 633 researches screened, we excluded 25 because they were non-English, 24 were book and book chapter, and 86 were conference paper, leaving a total of 498 articles for retrieval. We were able to find the full text of all articles, resulting in 498 articles for eligibility. At eligibility, upon reviewing the full text, we excluded another 3 articles which were identified to be non-empirical primary data. We also further excluded 303 articles because they were inconsistency with relevant background information. In addition, four articles whose study population was not higher education students and 158 articles whose study period was not post-COVID-19 would also be excluded. This left a final 30 articles in the final review sample for data analysis. Figure 1 further describes the process of inclusion/exclusion.

### 2.3 Extraction and analysis

We extracted the main study parameters into a Microsoft Excel literature grid consisting of multiple tabs (see Supplementary material). Data includes authors/year of publications, country of studies, study designs, study participants, data collection approaches, analysis methods, and results.

## 3 Findings and discussion

### 3.1 Descriptive analysis of articles

Of the 30 studies, 2 studies used female samples. The remaining 28 research samples included both female and male samples. The student samples in the study were aged between 18 and 27. The study population consisted of college student samples and teacher samples from different regions.

All studies were published in English. Ten of the studies were set in Asian contexts, followed by four studies set in English schools. Most studies have used both descriptive analysis and thematic analysis (Al-Amrani and Al-Ghaithi, 2023; Cradduck et al., 2022; Heilporn et al., 2023; Jeong, 2023; Walker and Koralesky, 2021; Yuyun, 2023; Zhou et al., 2023).

Among the 30 studies included in this review, quantitative research was the most commonly used approach, with 19 studies employed quantitative designs, 5 used qualitative designs, and 6 adopted mixed-methods designs. In terms of data collection methods, most studies (*n* = 16) used questionnaires only, while 6 combined questionnaires with interviews, and 2 relied solely on interviews. A total of 2 studies used interview method to collect data. In addition, one study adopted a case study, another combined it with a questionnaire, and 4 used experimental methods. Overall, questionnaires were the dominant data collection tool.

### 3.2 Research question 1: how is student engagement defined in the studies?

A number of articles expressed an intention to define student engagement within their abstracts, introductions, or main discussions. For example, student engagement was consisted of interacting with others (Cradduck et al., 2022), reflecting on the state of learning (Jones, 2022), and attitudes toward educators and peers (Vermeulen and Volman, 2024). We founded that each of the articles included in the review reflected some expressions of the definition of student engagement:

1. Researchers defined student engagement in three dimensions: behavioral, cognitive, and affective engagement.
2. Behavioral engagement refered to students’ willingness to participate in lessons, interact with peers, teachers, and the school environment. Cognitive engagement involved students’ capacity to plan, monitor, and reflect on their own learning processes. Affective engagement reflected students’ emotional responses, including their attitudes toward teachers and peers.

Next, we will discuss the definition of student engagement by drawing on literature from different settings. For behavioral engagement, Mohamed et al. (2023) noted “positive experiences include online interactions, course convenience, and teacher availability, while negative experiences include technology issues and feelings of confusion” (p.5).

According to Mohamed et al. (2023), student engagement was closely linked to student behavior. They found that accessible courses encouraged students to join learning activities to gain clearer and deeper knowledge. They also argued that better learning environments and improved technology increase behavioral engagement (Mohamed et al., 2023). Vrieling-Teunter et al. (2022) supported this view. They emphasized the role of social interaction regulation in course learning. Student behavioral engagement affects both learning outcomes and peer interactions. Limniou et al. (2022) focused on cognitive engagement, they defined it through three elements: learning goals, self-efficacy, and deep learning. For instance, Limniou et al. (2022) wrote:

…regarding cognitive engagement, the potential challenges could be related to potential distractions due to students’ study environment, which might lead to potential procrastination. On the other hand, the potential opportunities could be related to the effective use of time and online content to study in-depth their cognitive subject. (p.12)

In their study, Limniou et al. (2022) used two-way ANOVA and multiple regression analysis to examine students’ cognitive and behavioral engagement. They found that students’ behavior in online education may be influenced by their cognitive experiences. However, other researchers presented opposing views. For example, Su et al. (2024) pointed out that behavior may negatively affect cognition. In addition, for aspects of student affective engagement, Su et al. (2024) also stated that “previous studies have found that some key factors like learning interaction, self-regulation, and social presence could influence learning engagement and learning outcomes” (p.6).

In addition, the authors echoed the fact that the deeper analysis of student engagement can be centered on behavioral, cognitive, and affective engagement (Su et al., 2024). Students’ emotional changes and teachers’ attitudes have affected student engagement. Limniou et al. (2022) found that teachers’ positive emotional responses encouraged students to participate in online classes. Furthermore, students’ self-emotional regulation was also regarded as a potential consideration factor (Chiu, 2022).

Overall, the cognitive, affective and behavioral dimensions were indispensable perspectives for us to analyze student engagement. These dimensions are also closely interrelated. In the following section, we will discuss the impact of online education on student engagement.

### 3.3 Research question 2: what is the impact of online education on student engagement in higher education?

#### 3.3.1 Impact on the behavior of students

Currently, a wide range of e-learning platforms was available in online education (Wang et al., 2021). Platforms such as Microsoft Teams, Zoom, and Webex have become popular choices among teachers and students (Ünlüer, 2024). Its popularity was largely due to its flexibility and broad accessibility (Collazos et al., 2021a).

In the online education, students have experienced the novelty of participating in the online class. For example, Wut et al. (2024) found that first-year university students showed stronger motivation and engagement in new teaching methods. The students who have just entered university were more engaged in class (Wut et al., 2024). This may be because students tended to feel more eagerness and anticipation for their upcoming college-level knowledge. This engagement reflected their eagerness to learn college-level knowledge (Villarroel and González, 2022). Therefore, they showed greater willingness to adapt to the new instructional methods introduced by universities in the online learning environment (Collazos et al., 2021a).

Furthermore, prior studies have indicated that senior students and business majors exhibit higher levels of engagement in online learning (Collazos et al., 2021a; Villarroel and González, 2022; Wut et al., 2024). These students often expressed greater concern about entering the workforce due to uncertainties in future employment and social conditions (Wut et al., 2024). Students more frequently used online tools to review their learning and prepare for the future (Collazos et al., 2021a). Researchers also found that minority students and those working part-time were more likely to participate in online programs (Lu et al., 2022). Based on the above research, we believe that student engagement is influenced by various factors, including age, field of study, ethnicity, and region.

Prior research suggesed that the level of student engagement may progressively increase with age (Khan, 2021). This finding contrasts with Wut et al. (2024) who observed that first-year students showed higher levels of engagement. Khan (2021) explained that older students were generally more capable of adapting to new learning models and social environments, which may contribute to improved engagement.

Many online education platforms have adopted technological tools and instructional strategies to address student participation issues in virtual classrooms (Villarroel and González, 2022). For example, teachers used Moodle’s online education charts to track students’ classroom activity during online lessons (Laeeq et al., 2024). These tools helped teachers identify less active students and provide them with additional support (Khan, 2021). In addition, teachers frequently encouraged these students to participate in class discussions and collaborative tasks (Collazos et al., 2021a).

Although online education has become a widely accepted instructional mode in the Post-COVID-19 era, several challenges remain. In many underdeveloped regions, basic infrastructure remains insufficient to support online learning. Internet connections were slow and frequent disruptions hinder access to online platforms (Lu et al., 2022). These issues also negatively impact student’ ability to access online content.

#### 3.3.2 Impact on the cognition and emotion of students

In my opinion, modern online digital technologies have affected some students’ perceptions and learning experiences. These technologies have changed how students interact with peers and teachers. Some students participated less actively in collaborative tasks, classroom discussions, and student-teacher interactions during online learning (Ünlüer, 2024). Cognitive factors played an important role in this process (Chen et al., 2021). Others felt uncertain about their own academic abilities, which leads to reduced confidence when responding to teachers’ questions (Addae, 2023; Lasekan et al., 2024). The researchers need to think further about the relationship between behavior and cognition.

Most students experienced negative emotions during the COVID-19 era, which affected their cognitive engagement and learning behavior (Vu et al., 2022). Zhou et al. (2023) pointed out that some students turn off their microphones and webcams in online courses. These phenomena lead to students’ inability to absorb the e-learning materials provided by teachers (Zhou et al., 2023). It was also difficult to reflect on learning in a limited learning process. To address these challenges, educators emphasized the importance of supportive environments that foster critical thinking and problem-solving (Xinogalos, 2022).

As a response, many online programs began to align with students’ interests and goals (Chen et al., 2021; De Santos-Berbel et al., 2022). In countries such as China and regions in Latin America, institutions introduced flexible online models to meet diverse student needs (Lu et al., 2022). However, balancing engagement and monitoring in these evolving systems remained a major challenge for educators (Collazos et al., 2021a).

In many cases, the teaching of theoretical content was difficult to stimulate students’ interest (Vermeulen and Volman, 2024). If educators can increase emotional investment in online education, students may also be more willing to participate in the lesson. Teacher emotions influenced students’ sense of belonging, self-efficacy, and autonomy (Fredricks et al., 2004). A positive emotional climate enhanced motivation and supports academic engagement (Collazos et al., 2021b). Teachers can promote students’ learning by creating a good teaching atmosphere (Vermeulen and Volman, 2024). Neisser, in the field of cognitive psychology, mentioned that positive emotions such as interest and curiosity can significantly enhance learning, while negative emotions such as anxiety and frustration can pose a barrier (Neisser, 2014). Many first-year students started college online and never met their teachers or classmates in person (Wut and Xu, 2021). This made the already difficult transition to university life even more stressful (Yuyun, 2023). This has added new anxiety and difficulties for those students who have already been overshadowed by the epidemic (Kaoud et al., 2021). In my opinion, strengthening emotional connections in virtual classrooms may help students manage anxiety and improve their social and academic adjustment. These issues warrant further attention in future research.

## 4 Conclusion

The main objective of the study was to explore the intrinsic meaning of student engagement and to examine the impact of various factors on student engagement in online education during post-COVID-19. The findings indicate that changes in student behavior in the online education were significant and were related to cognition and emotion. These changes were important in shaping students’ future academic performance and development, as it revealed the dual nature of their physical and mental development. We have discovered the significant influence of emotional attitudes in online education. Han Yu, a prominent Chinese thinker from the Tang dynasty, once said, “A teacher is one who imparts moral principles, imparts knowledge, and resolves doubts.” This perspective suggests that the role of teachers is not only to deliver knowledge but also to cultivate students’ sense of self-efficacy. However, this study has several limitations. Only 30 articles from the Web of Science and Scopus databases were selected, and all data analyzed were secondary sources. Future research will expand the range of databases, including EBSCO and CNKI. In addition, primary data will be collected through interviews and surveys with faculty and students in higher education to allow for more in-depth investigation.
