# Supplementary material for: Exploring the impact of online education on student engagement in higher education in post-COVID-19: what students want to get?
Source: Front Psychol. 2025 Jul 9;16:1574886. doi: 10.3389/fpsyg.2025.1574886 (PMC12284282; doi:10.3389/fpsyg.2025.1574886)
Supplement: Supplementary file 1 [file Data_Sheet_1.pdf]

	Geographical Location	Title	Journal	Study Design	Research Participants	Data Collection Method	Data Analysis Techniques	Findings	Relevancy to my Review	Research Evaluation
1	Ghana, South Africa	Online student engagement in times of emergency: Listening to the voices of students	E-Learning and Digital Media	Qualitative Research	38 students who met the relevant study criteria	Case Studies	Thematic Analysis	Student Engagement, Online Learning, National Emergency, COVID-19, Higher Education, Ghana	High	Results of the selection of strategies for online student engagement through case studies. Students may have technical problems and administrative challenges in online education. A systematic discussion of student perceptions of methods to increase student engagement.
2	The Sultanate of Oman, Arabic	Enhancing blended learning quality: Perspectives of Omani university students during and beyond the COVID-19 pandemic	Language Teaching Research Quarterly	Mixed-Design Method	106 students (97 females, 9 males) studying in diverse faculties and different levels (Year 1 to Year 4), and their ages ranged from 18 to 27	Questionnaires and Semi-Structured Interviews	Descriptive Analysis & Thematic Analysis	The findings indicate that, overall, students expressed satisfaction with the quality of online learning and teaching materials in the blended learning context. However, some students expressed dissatisfaction with the online interaction and teacher feedback. Specifically, the absence of individualised teacher feedback seems to have negatively affected students' motivation to engage in practice activities and discuss them with teachers during face-to-face sessions	High	Students' perceptions of blended learning were analyzed through samples varying in age, gender, grade level, and major. A series of detailed descriptions of perceptions of online learning are developed in the article in particular. Among other things, the advantages and disadvantages of online education are considered from the students' perspective.The study's findings further support the role of blended learning in increasing satisfaction, enhancing learning outcomes, personalised studying experiences, collaboration, and instructor involvement.
3	Nanjing, China	Integrated media platform-based virtual office hours implementation for online teaching in post-COVID-19 pandemic era	KSII Transactions on Internet and Information Systems	Quantitative Research	140 undergraduate students using integrated media platform (IMP)	Questionnaires	Descriptive Analysis	The experiment results indicate that applying virtual office hours (VOH) in the Integrated media platform (IMP) can improve students' online presence and test scores. Furthermore, students'participating modes during VOH implementation can largely affect their degree of presence,which can be well classified by using principal component analysis	High	This paper designs an IMP to improve the interactions and even learning outcomes in postCOVID-19 pandemic era. In addition, this paper study the influence on students' presence in the onlinecourse 'Signals and Systems' when VOH is added in IMP.
4	Australia	University students' engagement with devices and technology: A comparison of pre-and post-Covid-19 student use	Journal of Telecommunications and the Digital Economy	Mixed-Design Method	<UniA>'s law and justice students; and <UniB>'s law and engineering students	Questionnaires and Semi-Structured Interviews	Descriptive Analysis & Thematic Analysis	Some participants had more experience with different technologies, there were limited differences between the cohorts' willingness to use, and their use of, technology for study purposes.	High	In the study for online technology college students conducted a study about online learning. College students with limited technology may be more enthusiastic about online education. Although the increased level of networking and technology in modern society has facilitated learning for college students, the extent to which students will utilize their mobile devices for learning remains a question, and ways need to be provided to improve student learning.
5	United Kingdom	Higher education students' perceptions of online learning during COVID-19—A comparative study	Education Sciences	Quantitative Research	the information of 512 participants could be used: 240 in Hungary, 141 in Wales and 131 in South Africa	Questionnaires	Descriptive Analysis	The research revealed significant differences between the participating universities students' experiences. The most important differences were in the 'home learning environment', followed by 'engagement' and the perception of 'impact on learning skills'.	High	Dimension-related questions helped explain students' different perceptions of the emergency online learning experience during the COVID-19 "lockdown" and explored different attitudes and self-reported behaviors.
6	Vilnius, Lithuania	Remote learning versus traditional learning: Attitudes of university students	Journal of Philosophy	Quantitative Research	180 full-time students	Questionnaires	Descriptive Analysis	The scientific analysis of computation data might allow drawing conclusions about students' preference: which mode of learning- remote or traditional - is beneficial and how much is each of them supportive.	High	The text explores the attitudes of university students towards distance and traditional English language teaching and raises issues that arise from utilizing distance learning. Among other things, students present technological issues related to online education that may give rise to associated learning pressures.
7	Madrid, Spain	Undergraduate student performance in a structural analysis course: Continuous assessment before and after the COVID-19 outbreak	Education Sciences	Quantitative Research	Student participation questionnaires for both school years	Questionnaires	Descriptive Analysis	The results highlight the robustness of the continuous assessment method used and emphasize the importance of having developed and validated online learning procedures to broach learning activities if contingency situations are required.	High	This study compared student performance using online continuous assessment in a structural analysis course for undergraduate architecture students before and after the COVID-19 outbreak. While recognizing the resilience of online education and the positives of applying a blended education model, it may not provide insight into the dynamics of student learning and student comprehension.
8	Quebec, Canada	Blended online courses: students' learning experiences and engaging instructional strategies	Pedagogies: An International Journal	Mixed-Design Method	a variety of disciplines and academic cycles at four Quebec universities (n = 482)	Questionnaires and Semi-Structured Interviews	Descriptive Analysis & Thematic Analysis	Following the analysis of student responses to open-ended questions, five recurring themes were identified: the enhancement of student-student interactions, the dynamism of synchronous and asynchronous sessions, the structured presentation of the course, the explanations and feedback from instructors, and the accessibility and involvement of instructors. For each theme, suggestions from student feedback are provided to facilitate student engagement in blended online courses.	High	The current state of affairs regarding student engagement in online courses is analyzed by comparing student surveys across majors as well as cycles of study. In the course of the study. Answers to questionnaires and related open-ended questions revealed the importance of course presentation and instructor's explanation in online education. The actual situation of student participation should also be analyzed.

9	Korea	Integrating technology into language teaching practice in the post-COVID-19 pandemic digital age: From a Korean English as a foreign language context	RELC Journal	Mixed-Design Method	The participants of this study were 300 university students in the central part of Korea	Questionnaires and Semi-Structured Interviews	Descriptive Analysis & Thematic Analysis	The result of this study revealed that the integration of social networks and mobile-assisted language learning (MALL) could support EFL university students' language learning experience and boost students' motivation and engagement in their learning process. This study also suggested that the concept of MALL could contribute to cultivating positive dynamics of classroom interaction.	High	The findings of the study highlight the benefits of incorporating technology in language education and the importance of adapting teaching methods to meet the evolving needs of learners in a rapidly changing post-COVID-19 pandemic digital landscape. The analysis of the positives of online education through the article contributes to the thoughtful analysis of the ideas associated with the mini-review.
10	Liverpool, United Kingdom	Relationships between undergraduate student performance, engagement, and attendance in an online environment	Digital Learning Innovations	Quantitative Research	31 students of mixed gender during the second year of their undergraduate study	the use of asynchronous lecture video viewing statistics	Descriptive Analysis	There is a positive correlation between increased on-site attendance and student achievement (a proxy for grades). The reasons for the disconnect between attendance and achievement in online and face-to-face environments are complex and contain many interrelated factors.	High	As there are many factors influencing the detection of online education attendance as well as participation, a systematic analysis of the relevant influencing factors is carried out in the article. The article presents findings from the perspective of virtual learning environments and discusses the opportunities and challenges.
11	Egypt	Online Learning in Egyptian Universities Post COVID-19 Pandemic: A Student's Perspective.	International Journal of Emerging Technologies in Learning (iJET)	Quantitative Research	The sample chosen was from the Schools of Business from three different universities in Egypt	Questionnaires	Descriptive Analysis	The main findings of this study revealed that the majority of students had reservations about online learning and that traditional learning (face-toface learning) was necessary for learning to occur (68%).	High	This study examined issues related to technological challenges, electronic communication, motivation for online learning, students' personal attributes and abilities, and online learning, motivation for online learning, students' personal attributes and abilities, and their interactions with colleagues and faculty. The findings of the study provide useful insights into students' attitudes towards currently adopted online learning methods. The results of the study provide useful insights into students' attitudes toward currently adopted online learning methods and suggest implications that could help educational institutions develop strategies to enhance the overall online learning experience.
12	United Kingdom	The impact of COVID-19 on UK higher education students: experiences, observations and suggestions for the way forward	Emerald Insight	Quantitative Research	349 UK higher education students	Questionnaires	Descriptive Analysis	Key findings suggest that the use of online virtual classrooms as a substitute for traditional face-to-face LTA has its positives and its negatives. The most significant positives are the "flexible assessments" and "digital content" and, in contrast, one of the significant drawbacks is the lack of interactions, this being true for both male and female students. However, as compared to females, males found to be missing "the campus life" more during the lockdown. Finally, the majority of student felt that there was a lack of support from the university and teaching staff during the lockdown. Universities' governance must take control of how this issue is driven forward and learn from the experience of students.	High	The variable of gender was taken into account in the research sample in this study. First, the students' perspectives were used to open a new debate on the methodology of long-term educational assistance during the epidemic. Second, the impact of the changes introduced as a result of the embargo on the students' experience was examined. Finally, strategies to be implemented by the management of higher education were proposed, based on the opinions of the students who participated in the study.
13	Pakistan	Integrated modular approach to provide optimized VLE for learners' engagement	Multimedia Tools and Applications	Quantitative Research	A selection of university students was made to help with the assessment of the suggested system	System Test Evaluation	Descriptive Analysis	This article proposes IMPROVE (Integrated Modular Approach to Provide Optimized VLE for Learners' Engagement), a modular-based strategy to enhance virtual learning environments (VLEs) for the Covid-19 pandemic scenario.	High	The proposed scheme significantly increases the constructive engagement of learners in online learning and has a favorable impact on learner performance and VLE usability. The proposed system solution is innovative and noteworthy, and it will significantly expand the knowledge domain.
14	United Kingdom	Student engagement, learning environments and the COVID-19 pandemic: A comparison between psychology and engineering undergraduate students in …	Education Sciences	Quantitative Research	psychology and EEE undergraduate students for the three undergraduate levels of studies	Questionnaires	Descriptive Analysis	Significant differences were found between the two learning environments in both faculties, with the majority of students perceiving that they were more engaged in the face-to-face environment (quantitative analysis). In addition, thematic analysis of students' qualitative feedback revealed that online student engagement was influenced by (1) behavior, (2) affect, (3) cognitive challenges (e.g., additional study load, lack of communication, and distractions in the home environment), and opportunities (e.g., effective use of study time and online content through interactive learning environments).	High	This study not only explored student engagement in online learning environments during the New Crown epidemic, but also attempted to explore potential differences in face-to-face learning environments in two faculties (Psychology and Electrical and Electronic Engineering). Student engagement in learning has been explored along three dimensions: cognitive, affective and behavioral. Students' academic performance has also provided valuable findings about students' preferences in face-to-face and online learning environments and has been considered as an indicator of students' course engagement.

15	China	Research on the influence of socially regulated learning on online collaborative knowledge building in the post COVID-19 period	Sustainability	Quantitative Research	participants from a comprehensive university in southwest China. A total of 77 students participated in the Digital Media Video Creation Course	Experimental method	Descriptive Analysis	The results showed that there was variability in the supply of SoRL intervention scripts and the actual selection status of the learners. The regulation foci activated in this study were time management, content monitoring, and atmosphere activation. Atmosphere activation drove collaborative learning activities to continue. Time management and content monitoring drove collaborative knowledge building (CKB) to a deeper level.	HIgh	This study analyzes the limitations of online education through relevant experiments.This study is of great significance in revealing the impact mechanism of socially regulated learning (SoRL) on collaborative knowledge building (CKB).
16	Saudi Arabia	Lived Experience: Students' Perceptions of English Language Online Learning Post COVID-19.	Journal of University Teaching and Learning Practice	Quantitative Research	260 students were surveyed online	Questionnaires	Descriptive Analysis & Thematic Analysis	The findings of this study indicate that successful online learning outcomes in international educational settings require the incorporation of digital technologies and synchronous teaching methods.	High	The results indicate that students, especially males, find online learning more effective than traditional learning despite its limitations and prefer synchronous audio and video learning. The study emphasizes the need to consider students' demographics and digital equity in the competitive higher education market and recommends exploring the perceptions of larger student samples from different majors. Online learning offers convenience, flexibility, and easy access to study resources, with previous research indicating its growing popularity worldwide.
17	China	Looking back to move forward: comparison of instructors' and undergraduates' retrospection on the effectiveness of online learning using the nine-outcome influencing factors	Humanities & Social Sciences Communications	Qualitative Research	46 higher education students and 18 higher education teachers	Reflective Diaries & Semi-Structured Interviews	Thematic Analysis	The research revealed that instruction ranked highest among the 9 factors, followed by engagement, self-regulation, interaction, motivation, and others. Moreover, teachers and students had different attitudes toward instruction. Thirdly, teacher participants were different from student participants given self-efficacy and self-regulation due to their variant roles in online instruction. Lastly, the study reflected students were not independent learners, which explained why instruction ranked highest in their point of view.	High	This study delves into the retrospections of undergraduate students concerning their online learning experiences after the COVID-19 pandemic, using the nine key influencing factors: behavioral intention, instruction, engagement, interaction, motivation, self-efficacy, performance, satisfaction, and self-regulation.
18	Istanbul	Challenges in online learning environments: from the perspective of hearing-impaired university students and their lecturers	Problems of Education in the 21st Century	Qualitative Research	20 hearing-impaired students and 19 faculty members as participants	Interviews, Open-ended Questionnaires, and the Researcher's Journal	Thematic Analysis	Findings from the study highlighted a multitude of challenges encountered in the online learning process, affecting both faculty members and students at the School for the Handicapped (SfH). The challenges encountered by participants in online learning at the SfH were found to be related to difficulty in understanding lessons, lack of subtitles and sign language translation, difficulty accessing computers and software, Internet problems, low camera usage, low motivation, and lack of interaction.	High	This study has shown that in online education during the Covid-19 pandemic, hearing impaired college students and their lecturers at the SfH face various challenges. It was seen that the challenges were caused by the faculty members, hearing-impaired students and the learning management system used in distance education. In addition to the limitations in the language and communication skills of d/hh students, the lack of learning due to the lack of motivation caused by being away from face-to-face education in the classroom environment emerged as the biggest problem in online education.This has implications for mini-review related content.
19	Chile	Students' learning perception in engineering, health and education during emergency remote education in Chile	BIBLIOTECE I UDD	Quantitative Research	a sample of 929 higher education students regarding their ERE experience	Questionnaires	Descriptive Analysis	Education students showed a more positive perception of learning, used a deep learning approach, and rated more positively the empathy shown by their professors and the possibilities for class participation than other students. Engineering students had the lowest scores on perceived learning and on almost all other variables. In general, students with a better perception of learning presented a higher engagement in their studies and a lower use of surface learning approach.	High	This paper discusses the problems that may exist regarding online education and highlights the key influences that teachers have on their students.Relational factors that depend on teachers were also strongly relevant, such as promoting participation and dialogue with and among students and the empathy and affective bonding they show, especially in crises.
20	the Netherlands	Promoting student engagement in online education: online learning experiences of Dutch university students	Technology, Knowledge and Learning	Qualitative Research	via Zoom with students (N = 25) from different social sciences programs at the University of Amsterdam	Online Semi-structured Focus Groups	Thematic Analysis	Findings reveal that synchronous and asynchronous online learning activities stimulate three dimensions of engagement and their underlying mechanisms. Behavioral engagement was stimulated through activities that promoted attention and concentration, stimulated effort, broke down barriers, and provided flexibility. Emotional engagement is stimulated through activities that promote group feelings, encourage interaction, and create empathy and trust. Cognitive engagement is stimulated through activities that provoke discussion and personalization. This study provides instructors with insights into how to promote student engagement in online education.	High	This study identifies learning activities and their underlying mechanisms that promote students' behavioral, affective, and cognitive engagement in an online learning environment. Behavioral engagement is enhanced through mechanisms that enhance attention and focus, stimulate effort, break down barriers, and provide flexibility. For most students, affective engagement was elicited through mechanisms that promote group emotions, facilitate interaction, and build empathy and trust. Finally, cognitive engagement is facilitated through mechanisms that provoke discussion (in synchronous activities) and personalization (in asynchronous activities).

21	the Netherlands	Facilitating peer interaction regulation in online settings: The role of social presence, social space and sociability	Educational Psychology	Quantitative Research	41 students (32 female, 9 male)	Questionnaires	Descriptive Analysis	The results suggest that the planning stage is the most important stage in supporting students' sense of social presence, as it is during this stage that the regulation of peer interactions becomes important. Sociality also plays an important role here, but its importance decreases over time during self-regulation. In subsequent stages of social learning, students look for alternative ways to increase their social presence and social space in order to shape the regulation of peer interactions from a trust perspective. During the assessment phase, students realize the importance of social presence but are less aware of the importance of social space for peer interaction regulation.	High	This study suggests that the social nature of current virtual learning environments (VLEs) is insufficient to support social presence and the social spaces that come with it, and is therefore not conducive to the specification of peer interactions in online courses. For this reason, the planning phase is particularly important, as this is usually the moment of connection with classmates. This study mentions ways and means regarding affective interventions that can be quite helpful for students' motivation to participate.
22	Ho Chi Minh City, Vietnam	Synchronous Online Learning in Higher Education	Journal of Ethnic and Cultural Studies	Quantitative Research	475 Vietnamese college studen	Questionnaires	Descriptive Analysis	Our research showed that when engaged in SOL, the higher education research participants had a positive learning experience, perceived growth, and received learning assistance, in response to our quantitativ examination of exploratory factor analysis and our qualitative counterpart of theme-based analysis.	High	Online education is undoubtedly a new mode of receiving knowledge for students. They have considerable confidence in it, but show geographical limitations in the face of different cultural backgrounds, such as Internet problems.
23	Canada	Student and instructor perceptions of engagement after the rapid online transition of teaching due to COVID-19	Natural Sciences Education	Mixed-Design Method	Fifteen courses—including laboratory, discussion-based, large lecture, tutorial, and problem-based learning—within a multi-disciplinary faculty at a large research-intensive Canadian university	Questionnaires and Open-ended Interviews	Descriptive Analysis & Thematic Analysis	Students found synchronous activities more engaging. Students experienced positive and negative outcomes related to classroom engagement when transitioning rapidly to online learning during a global pandemic	High	This study identified what was enhanced or diminished regarding student engagement and found that affective engagement was diminished while cognitive engagement was enhanced. Students found synchronous activities more engaging. It can be found in the study that the grade level of the students affects the motivation to participate to a great extent.
24	United States of America	Do playfulness and university support facilitate the adoption of online education in a crisis? COVID-19 as a case study based on the technology acceptance model	Sustainability	Quantitative Research	A structural equation model of participation intention is constructed, and 342 valid samples are obtained by questionnaire survey.	Questionnaires	Descriptive Analysis	It was found that enhancing students' attitudes toward participation, perceived ease of use, usefulness, and fun, as well as strengthening university support all contributed to optimizing students' willingness to participate in online education.	High	This study explored the influencing factors of college students' willingness to participate in online education based on the technology acceptance model, and found that the direct factors affecting the willingness to participate in online education are the attitude of participation and perceived interestingness.
25	Hong Kong, China	Engaging university students in online learning: a regional comparative study from the perspective of social presence theory	Journal of Computers in Education	Quantitative Research	495 university students in Mainland China	Questionnaires	Descriptive Analysis	In all regions, a consistent positive correlation was found between satisfaction and academic achievement. Malaysia is unique compared to Mainland China and Hong Kong in that no association was found between social background and online communication with student engagement.	High	This study investigates the factors influencing university students' online learning engagement from three distinct aspects, namely, behavioural, cognitive and emotional engagement.The process of analyzing the relationship between the three involves relevant influences concerning the impact on student engagement and emphasizes the important role of cognitive affect in education.
26	Hong Kong, China	Person-to-person interactions in online classroom settings under the impact of COVID-19: a social presence theory perspective	Asia Pacific Education Review	Qualitative Research	in-depth interviews with 17 university students and 7 instructors	In-depth Interview Approach	Thematic Analysis	Studies have highlighted that the lack of person-to-person interactions threatens the quality of online education. It found that student-to-instructor and student-to-student interactions cannot fully establish cognitive social presence and affective social presence. It then provided recommendations including encouragement, incentives, breakout rooms, and engagement techniques.	High	In the article, the importance of teacher-student interaction in online education is emphasized, and problems and challenges are analyzed from both the teacher's and the student's perspectives. Differences in gender, specialty, and teaching age were included in the sample. It was found that challenges to student-instructor interactions included (1) feedback, response, and clarification; (2) recognition of student participation; and (3) social presence and other factors, while barriers to student-student interactions included (1) teamwork, (2) learning from peers, and (3) social presence.
27	Thessaloniki, Greece	Moving from face-to-face to online learning in a week due to the COVID-19 pandemic: higher education students' perceptions	International Journal of Technology Enhanced Learning	Quantitative Research	71 undergraduate students and 39 postgraduate students	Questionnaires	Descriptive Analysis	Students evaluated positively the quality, organization and presentation of synchronous online courses, but they were divided as to whether their quality is equivalent to that of face-to-face courses. The most prominent problems refer to the quality of Internet connection, lack of appropriate infrastructure especially for online lab courses, lower quality communication and interaction and a difficulty in keeping concentrated on the course. Despite these issues, instructors and students embraced successfully the solution of online learning.	High	Concluding, it turned out that both undergraduate and postgraduate students viewed positively the transition from face-to-face to online learning, but at the same time they highlighted aspects of the educational process that are far more effective in a face-to-face context. Proposing and empirically investigating ways for dealing with troublesome aspects of online learning is important for delivering more effectively online courses. Finally, an interesting finding of this study was the indication that postgraduate students seem to be much more thoughtful than undergraduate students for the degree that the quality of online courses and even more online labs can be considered equivalent to the quality of face-to-face courses and labs.

28	Indonesia	Investigating university student engagement in online learning: A case study in EFL classroom	Indonesian Journal of Applied Linguistics	Mixed-Design Method	26 university students in a sample of EFL teaching in an Indonesian context	Case Studies & Questionaires	Descriptive Analysis & Thematic Analysis	This study implies that synchronous and asynchronous activities should be incorporated into online learning to get more engaging interaction among students.	High	The study encompasses five elements of online engagement: social engagement, cognitive engagement, behavior engagement, collaborative engagement, and emotional engagement. The prominent finding has revealed that university student engagement in online learning has different significance levels. Most students are more involved in synchronous online activities as they can interact with their professors and peers in real time. Synchronous and asynchronous learning provide substantial and comparable results.
29	the Netherlands	Influence of online collaborative learning on social network and academic performance of medical students: lessons learned from the COVID-19 pandemic	Frontiers in Medicine	Quantitative Research	102 students participated before and 167 students during the pandemic	Questionnaires	Descriptive Analysis & Thematic Analysis	The results showed that the size of students’ social networks during the pandemic was significantly smaller than before the pandemic. Besides, the formation of social networks differed between first- and second-year students, and between domestic and international students. However, academic performance did not decline during the COVID-19 pandemic.	High	When institutions implement learning with social distancing, such as online learning, they need to consider changes in students’ social networks and provide appropriate support.The results showed that the size of students’ social networks was smaller during than before the COVID-19 pandemic, especially their friendship network. First-year students perceived more severe challenges than second-year students, and international students experienced a greater impact of the pandemic than domestic students. Although students faced difficulties in social network formation during the pandemic, their academic performance was higher than that of students in the before-pandemic cohort. When institutions are forced to implement blended or online learning during, for instance, pandemics or other situations of force majeure, they need to ensure to create a conducive learning environment by considering social, educational, and mental support to compensate for shortcomings caused by shrinking social networks of their students.
30	Indonesia	Investigating factors influencing students' engagement in sustainable online education	Sustainability	Quantitative Research	452 university students to identify the factors that influence students’ participation during online classes	Questionnaires	Descriptive Analysis	It is worth mentioning that a significant proportion of students, specifically 61.7%, demonstrated a considerable degree of engagement with faculty content due to its high quality. With respect to the role of social presence, possession of a designated private space boost (69.2%) and requiring students to use their webcams are found to be critical for students’ engagement. Lastly, teaching presence as a factor in enhancing students’ engagement is demonstrated by promoting interactive classrooms and providing rapid feedback to students by teachers. The findings demonstrate the importance of teachers’ quality course material, interactive classes, and the benefits of a physical environment free from distractions for optimizing students’ engagement.	High	Teacher content as a cognitive factor has a significant impact on promoting student engagement in online classes. The study confirms the importance of private classrooms as a social factor in increasing student engagement in online classes. The findings emphasize the importance of instructional presence in increasing student engagement in online courses. Sustained student engagement in the online classroom is closely related to the development of cognitive, social, and instructional presence.
Integrated Analysis of Literature Review:<br>Online education is both an opportunity and a challenge for the development of today's education. Judging from the 30 articles retrieved through keywords, almost all of them also mentioned similar viewpoints. During post-covid-19, online education was widely used for teaching by schools and institutions due to its efficiency, cross-regional nature and abundant resources. However, there are still some challenges, such as network issues and teaching mode problems. The opportunities and challenges brought by online education have a certain impact on student participation. Among the collected articles, the discussions on cognition, emotion and behavior have different voices. The emergence of technical problems will cause students to experience a great deal of learning pressure, which in turn will affect students' behavioral participation. This is the case in terms of universality, but students with strong learning initiative will still find ways to solve problems. Besides, most students have online classes at home. The parenting style of parents is also a factor that can be considered. On the other hand, the vast majority of the literature mentions the importance of emotional engagement. Based on our own teaching practice, we have also discovered similar viewpoints. Teachers' emotions and attitudes can enhance students' sense of self-efficacy and gain. Students' classroom participation is on the one hand self-control and on the other hand the influence from the environment. Han Yu, a renowned educator in China, once said, "A teacher is one who imparts moral principles, imparts knowledge and resolves doubts." The responsibility of teachers is not only to impart knowledge, but also to constantly influence students' sense of efficacy. Stimulate students' potential through emotional participation. The role of blended education has also been seen in different articles. This is also a direction that online education can consider in its future development. Cognitive factors are also factors that need to be considered. For student participation, it is accomplished based on certain cognitive experiences. Influence students' behavioral participation in online education through the integration of cognition and emotion. We responded to the specific analysis in the article.										
* The order in which the articles are listed in the table is randomized and not sequential.										
